# Potential Impact of miR-137 and Its Targets in Schizophrenia

**DOI:** 10.3389/fgene.2013.00058

**Published:** 2013-04-26

**Authors:** Carrie Wright, Jessica A. Turner, Vince D. Calhoun, Nora Perrone-Bizzozero

**Affiliations:** ^1^Department of Neurosciences, Health Sciences Center, University of New MexicoAlbuquerque, NM, USA; ^2^The Mind Research NetworkAlbuquerque, NM, USA; ^3^Psychology Department, University of New MexicoAlbuquerque, NM, USA

**Keywords:** miRNA, schizophrenia, miR-137

## Abstract

The significant impact of microRNAs (miRNAs) on disease pathology is becoming increasingly evident. These small non-coding RNAs have the ability to post-transcriptionally silence the expression of thousands of genes. Therefore, dysregulation of even a single miRNA could confer a large polygenic effect. Schizophrenia is a genetically complex illness thought to involve multiple genes each contributing a small risk. Large genome-wide association studies identified miR-137, a miRNA shown to be involved in neuronal maturation, as one of the top risk genes. To assess the potential mechanism of impact of miR-137 in this disorder and identify its targets, we used a combination of literature searches, ingenuity pathway analysis (IPA), and freely accessible bioinformatics resources. Using TargetScan and the schizophrenia gene resource (SZGR) database, we found that in addition to CSMD1, C10orf26, CACNA1C, TCF4, and ZNF804A, five schizophrenia risk genes whose transcripts are also validated miR-137 targets, there are other schizophrenia-associated genes that may be targets of miR-137, including ERBB4, GABRA1, GRIN2A, GRM5, GSK3B, NRG2, and HTR2C. IPA analyses of all the potential targets identified several nervous system (NS) functions as the top canonical pathways including synaptic long-term potentiation, a process implicated in learning and memory mechanisms and recently shown to be altered in patients with schizophrenia. Among the subset of targets involved in NS development and function, the top scoring pathways were ephrin receptor signaling and axonal guidance, processes that are critical for proper circuitry formation and were shown to be disrupted in schizophrenia. These results suggest that miR-137 may indeed play a substantial role in the genetic etiology of schizophrenia by regulating networks involved in neural development and brain function.

## Introduction

First discovered in *C. elegans* (Ruvkun et al., 2004), miRNAs are small non-coding RNA sequences that play a significant role in the regulation of gene expression, particularly at the post-transcriptional level. Regulation by miRNAs is a complex process, in which some miRNAs are capable of targeting and repressing hundreds to even thousands of transcripts (Selbach et al., 2008). Additionally, many mRNAs are targeted by several miRNAs (Selbach et al., 2008; Hu, 2009). It is estimated that the expression of at least 30% of human genes are regulated by miRNAs (Lewis et al., 2005; Selbach et al., 2008). With such high levels of potential regulatory influence, some miRNAs may have enormous impact on gene expression and such an impact may play a role in the pathophysiology or etiology of diseases with an elusive genetic basis, such as schizophrenia. This disease is genetically complex and very little is understood about its genetic basis or underlying mechanisms (Hamshere et al., 2012). Several recent lines of research suggest that miRNAs may be involved. First, the gene encoding the DiGeorge syndrome critical region gene 8 protein (DGCR8), one of the components of the nuclear miRNA processing complex, is located in a chromosomal location (22q11.2) associated with high risk for schizophrenia (Stone et al., 2008). Also, a single-nucleotide polymorphism (SNP) in the gene for a particular miRNA, miR-137 was found to be one of the common alleles associated with schizophrenia (Kwon et al., 2011; Ripke et al., 2011). This report examines the role of this miRNA in brain development and function and explores the potential functional impact of its known and putative targets on schizophrenia. By identifying the putative and validated miR-137 targets, and examining their potential contribution to functional networks, we hope to shed more light on the possible role of this miRNA in the etiology of the disease.

## microRNA

microRNAs (miRNAs) are small non-coding RNAs with the ability to silence the expression of multiple target genes by binding to specific sequences of mRNAs. A single miRNA can impact hundreds to thousands of targets and can affect pathways controlling a large variety of processes, from normal development to oncogenesis. Pairing is primarily based on sequence complementarity of a “seed” sequence within the miRNA to a binding site of the mRNA, generally in the 3′ UTR of the mRNA being suppressed (Bartel, 2009). The mechanisms by which miRNAs suppress gene expression are still not well elucidated; however mRNA destabilization and translational repression have been demonstrated as dominant methods of repressing subsequent protein expression (Carthew and Sontheimer, 2009).

**Figure 1:**
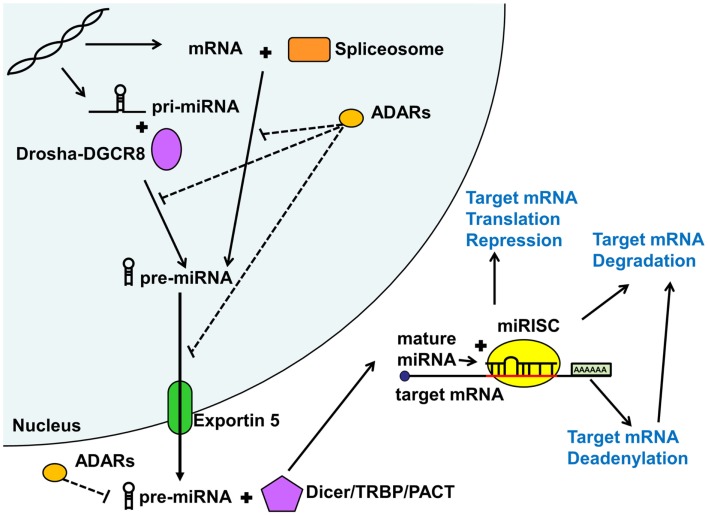
**MicroRNA biogenesis and function**. MicroRNAs (miRNA) are generated via the mirtron or canonical pathway. Primary microRNA (pri-miRNA) from the canonical pathway is further processed by the Drosha-DGCR8 complex into precursor miRNA (pre-miRNA). Pre-miRNAs derived from both pathways are then exported from the nucleus by Exportin 5. In the cytoplasm, this pre-miRNA is further processed by the Dicer/TRBP/PACT complex into a duplex miRNA. One of the strands (guide strand or mature miRNA) is then loaded into the miRISC complex along with the target mRNA. Depending on the degree of sequence complementary, miRNAs lead to translational repression, deadenylation, or degradation of the target mRNAs, all reducing downstream protein expression. Adenosine deaminases acting on RNA (ADARs) may inhibit various steps of miRNA processing thus reducing or shifting miRNA function. See text for more detail.

As shown in Figure 1, miRNAs are either first transcribed in the nucleus as primary miRNAs (pri-miRNAs) or less commonly spliced from introns (Lin et al., 2006). The pri-miRNAs are then processed in the nucleus by the microprocessor complex involving the RNAse III enzyme Drosha complexed with the targeting protein DGCR8 into approximately 89 nucleotide long sequences termed pre-miRNAs (Lindow and Gorodkin, 2007; Carthew and Sontheimer, 2009; Cuperus et al., 2011). Intron derived miRNAs termed mirtrons are excised by the RNA spliceosomal components and do not require further processing. These pre-miRNAs are then exported out of the nucleus by Exportin 5 (Lindow and Gorodkin, 2007) and further processed in the cytoplasm by a protein complex containing Dicer and its associated proteins the *trans*-activation response RNA binding protein (TRBP) and the protein activator of the interferon induced protein kinase (PACT) into an approximately 22 nucleotide long double stranded RNA (Carthew and Sontheimer, 2009). These double stranded sequences then separate and a single strand termed guide strand (mature miRNA) is loaded with a complementary mRNA into an Argonaute containing microRNA-induced silencing complex (miRISC) where the miRNA ultimately binds the target sequence (Carthew and Sontheimer, 2009) to repress ensuing protein expression.

Adenosine deaminases acting on RNA (ADARs) can also modify and regulate miRNA function, both in the nucleus and cytoplasm (Figure 1). Adenosine deaminases can modify miRNA processing and function not only by editing pri- and pre-miRNA sequences but also through steric hindrance in the absence of RNA editing (Heale et al., 2009). Alterations in miRNA sequence can lead to shifted (Kawahara et al., 2007), reduced, or eliminated targeting or reduction in mature miRNA production (Luciano et al., 2004). ADAR miRNA editing seems to play an important role in mammalian brain development (Ekdahl et al., 2012). Surprisingly, very few studies have addressed the potential role of ADAR mRNA editing in psychiatric illness, with conflicting results (Silberberg et al., 2012; Zhu et al., 2012). However, given that the studies only addressed mRNA editing effects, there may be an alternative large scale regulatory impact of ADARs on miRNA regulation of gene expression, which remains to be explored.

## Schizophrenia and miRNAs

Schizophrenia is a severe mental illness that has an average lifetime development risk of 0.7%, with an average annual incidence rate of 15 per 100,000 (Tandon et al., 2008; Blanchard et al., 2011). Heritability of the disease is estimated at 81% based on twin studies (Sullivan et al., 2003). The term schizophrenia meaning, “split mind” was coined by Bleuler in 1911 to describe the dissociation of thought, ideas, identity, and emotion that characterize the illness (Moskowitz and Heim, 2011). The disease is described by negative symptoms of social withdrawal, positive symptoms of psychosis including hallucination and delusion, cognitive impairment, and in some cases mood dysregulation (van Os and Kapur, 2009). These symptoms lead to secondary disparities in health and premature mortality, stressing the need for better understanding of the underlying mechanisms.

This mental illness is largely heterogenetic, with different patients having different associated genetic alterations and varied symptomology (Sebat et al., 2009; Green et al., 2012; Wahlsten, 2012). Evidence also suggests that it is a polygenic disorder, in which many common genetic variants may each contribute a small increased risk (Purcell et al., 2009). Given this evidence for such polygeneity and the fact that single miRNAs have the potential to regulate hundreds to thousands of transcripts (Selbach et al., 2008), it seems plausible that disruption of a miRNA could lead to abnormal expression levels of many genes, which could in turn contribute to schizophrenia vulnerability. Many miRNAs were found to be differentially expressed in blood samples and post-mortem tissue of patients with schizophrenia (Perkins et al., 2007; Beveridge et al., 2010; Kim et al., 2010; Gardiner et al., 2011; Lai et al., 2011; Moreau et al., 2011; Santarelli et al., 2011; Miller et al., 2012) providing further evidence that miRNAs may impact the disorder.

One miRNA of particular interest is hsa-miR-137. The recent study from The Schizophrenia Psychiatric Genome-Wide Association Study (GWAS) Consortium (Ripke et al., 2011), with an initial sample size of 21,856 and a replication sample of 29,839 found several new significant loci to be associated with schizophrenia, the strongest association being the SNP rs1625579 within an intron containing the primary transcript of miR-137. Several of the other associated loci contained predicted targets of miR-137 (Ripke et al., 2011) supporting evidence that miR-137 might play a role in the disorder. A subsequent smaller GWAS study examining a smaller sample of patients with treatment-resistant schizophrenia from the UK receiving clozapine treatment, also found significant association for CACNA1C but did not replicate the finding for CSMD1 or miR-137, yet when the data was grouped with the earlier GWAS study both were found to be significant (Hamshere et al., 2012).

Besides GWAS studies, two additional experimental approaches are beginning to shed light onto the possible link between miR-137 and schizophrenia. First, using luciferase reporter assays, Kwon et al. (2011) confirmed that transcripts of other genes associated with schizophrenia in the largest GWAS study (Ripke et al., 2011), namely CSMD1, C10orf26, CACNA1C, and TCF4 can in fact be regulated by miR-137. Likewise, Kim et al. (2012) recently demonstrated that ZFN804A another gene highly associated with the illness (O’Donovan et al., 2008; Steinberg et al., 2011) can also be silenced by miR-137 *in vitro*. While further research is required to confirm the association of the SNP in miR-137 with risk for schizophrenia, the findings that miR-137 can regulate the expression of other schizophrenia-associated genes provides new clues on how miR-137 may play a role in the illness. Along these lines, a recent post-mortem tissue study suggests that the T risk allele of rs1625579 may be associated with decreased miR-137 expression in the dorsolateral prefrontal cortex of patients (Guella et al., 2012), further suggesting a potential dysregulation of the miRNA’s targets.

## Role of miR-137 in Brain Development and Function

miR-137 is expressed in embryonic and adult brains (Sun et al., 2011) and was found to be highly enriched in synaptosomes from P15 rats (Siegel et al., 2009). Using *in situ* hybridization, high expression of this microRNA was observed in the dentate gyrus, an area highly active in adult neurogenesis (Smrt et al., 2010). The involvement of this miRNA in neuronal development was confirmed by several functional experiments. Overexpression of miR-137 was shown to decrease proliferation of mouse embryonic neural stem cells leading to their premature neuronal differentiation (Sun et al., 2011) and similar effects were observed in adult mouse neural stem cells derived from the subventricular zone or from brain tumors (Silber et al., 2008). However, overexpression of miR-137 in the adult neural stem cells derived from the subgranular layer of the dentate gyrus was found to disrupt the expression of stage specific differentiation markers such as DCX and NeuN as well as dendritic arborization in these newly generated neurons (Smrt et al., 2010). This apparent discrepancy is likely due to distinct roles of miR-137 in different stages of neuronal differentiations as well as the intrinsic properties of cells in different neurogenic zones. In a recent study, overexpression of miR-137 was shown to decrease maturation and increase proliferation while antagonism of miR-137 in adult neural stem cells increased neuronal differentiation and reduced proliferation (Szulwach et al., 2010). These experiments suggest that a balanced expression of miR-137 is necessary to maintain appropriate neuronal differentiation and proliferation and thus regulate neurogenesis. Given the important role that neurogenesis plays in learning, memory, and mood regulation, disruption of these essential functions may have significant effects that could lead to some of the symptomology seen in schizophrenia (DeCarolis and Eisch, 2010).

## Association of miR-137 and Cognitive Function

Recent genetics imaging studies have also found a correlation of miR-137 with schizophrenia. Firstly, utilizing gene set enrichment analysis to assess the contribution of gene regulatory networks to the illness, Potkin et al. (2010) found that miR-137 was implicated in two individual GWAS imaging genetics studies of patients performing the Sternberg item recognition paradigm (SIRP) working memory task (Potkin et al., 2009a,b). Given the findings of the large 2011 GWAS study (Ripke et al., 2011), a subsequent study examining subjects at risk for schizophrenia and bipolar disorder differentiated their cohorts based on this SNP (Whalley et al., 2012). Subjects with two copies of the “T” risk allele were assigned as risk positive and those carrying one or no copies of the allele were classified as risk negative subjects. A reduced response in the right posterior medial frontal gyrus region to increasingly difficult sentence completion tasks was noted only in the risk positive group across all the groups: schizophrenia at risk, bipolar at risk, and controls. This suggests that miR-137 may have a general effect on executive function. Also, schizophrenia at risk subjects had differential activation of the left amygdala and left pre/postcentral gyrus, suggesting a more schizophrenia-specific effect of the allele as well (Whalley et al., 2012).

Recent studies also examined the role of this miRNA on other functional endophenotypes. The risk allele in miR-137 was potentially associated with the P300 endophenotype in schizophrenia patients (Decoster et al., 2012). In addition, when combined with greater negative symptoms, the rs1625579 SNP genotype predicted membership of patients in a subgroup with severe cognitive deficits (Green et al., 2012). In this study, cognitive functioning was evaluated by a battery of tests such as the Letter-Number Sequencing Test to assess working memory, and the Controlled Oral Word Association Test to assess executive function. First patients were categorized based on their performance with this battery of tests into either a cognitive deficit group or a cognitive spared group. Patients with more severe negative symptoms and the “G” protective allele were surprisingly more likely to have been previously grouped in the cognitive deficit group (Green et al., 2012). Another study examining carriers of the risk allele among psychosis patients, including those with schizophrenia, schizoaffective disorder and bipolar affective disorder I, found that carriers, particularly those homozygous for the “T” risk allele, had lower scores for psychotic symptoms and a subtle deficit in performance of episodic memory and attention control tasks (Cummings et al., 2013).

While studying the clinical effects of chromosome 1p21.3 microdeletions, the region containing the MIR-137 gene, Willemsen et al. (2011) found an association with intellectual disability and autism disorder spectrum-like behavior. Furthermore, lymphoblastoid cell lines from these patients were found to have reduced levels of miR-137 and enhanced levels KLF4 and the previously verified target genes MITF and EZH2. The authors also confirmed that miR-137 is highly expressed in the hippocampus, occipital cortex, and frontal cortex in human post-mortem tissue, as well as in the synaptosomal fractions in mouse brain preparations, providing further evidence that miR-137 may play a role in synapse formation during brain development and function.

## Targets of miR-137

To further understand the possible mechanism of miR-137 in schizophrenia, we used available databases and the literature to identify putative and validated targets. Using the list of potential and experimentally verified targets, we then evaluated their chromosomal location and temporal patterns of expression. Finally, we examined how these targets cluster within biological pathways to identify which functions would be affected if miR-137 levels were dysregulated as shown by initial post-mortem tissue studies (Guella et al., 2012).

### Putative targets

Putative targets were identified by querying the publically available TargetScan Human release 6.2 database updated June 2012 for hsa-miR-137 (Friedman et al., 2009). Selecting for target genes respective to site conservation resulted in 1144 putative target genes. The Ensemble cytoband location for each gene was identified by querying for each gene offered in the freely available GeneCards encyclopedia at www.genecards.org (Stelzer et al., 2011) (Table S1 in Supplementary Material). All subsequent analyses were performed using this list, as TargetScan offers several advantages over other target prediction algorithms given its unique consideration for sequence context in addition to conservation and seed sequence complementarity (Grimson et al., 2007; Friedman et al., 2009). This list was then evaluated to identify targets studied for experimental validation in the following section.

Examining genes that may play a role in schizophrenia, we compared our putative target lists against a schizophrenia-associated gene list of 278 protein-coding genes from the publicly available schizophrenia gene resource (SZGR), a database of a variety of schizophrenia related gene lists (Jia et al., 2010). We chose to use the association studies gene list that is derived from the SchizophreniaGene (SZGene) database and further evaluated for consistency across studies using a combined odds ratio (OR) method (Sun et al., 2008). Of the 1144 TargetScan putative target list, 25 genes intersected with the SZGR schizophrenia-associated gene list. These genes are listed in Table 1, including cytoband location information identified from GeneCards. Estimating that there are 20,000 genes in the genome, and that 278 are considered risk genes for schizophrenia, the probability that a randomly chosen sample of 1144 genes contains 25 or more of these risk genes is 0.017. Therefore, this result suggests that miR-137 targets are enriched in schizophrenia risk genes.

**Table 1:** SZGR associated miR-137 target genes*.

Gene symbol	Full name	Cytoband
ACSL6	Acyl-CoA synthetase long-chain family member 6	5q31.1
ATXN1	Ataxin 1	6p22.3
BRD1	Bromodomain containing 1	22q13.33
C18orf1	Chromosome 18 open reading frame 1	18p11.21
CHGA	Chromogranin A (parathyroid secretory protein 1)	14q32.12
ERBB4	V-erb-a erythroblastic leukemia viral oncogene homolog 4 (avian)	2q34
FOXP2	Forkhead box P2	7q31.1
FXYD6	FXYD domain containing ion transport regulator 6	11q23.3
FZD3	Frizzled homolog 3 (*Drosophila*)	8p21.1
GABRA1	Gamma-aminobutyric acid (GABA) A receptor, alpha 1	5q34
GRIA1	Glutamate receptor, ionotropic, AMPA 1	5q33.2
GRIA4	Glutamate receptor, ionotropic, AMPA 4	11q22.3
GRIN2A	Glutamate receptor, ionotropic, *N*-methyl-d-aspartate 2A	16p13.2
GRM5	Glutamate receptor, metabotropic 5	11q14.3
GSK3B	Glycogen synthase kinase 3 beta	3q13.33
HTR2C	5-Hydroxytryptamine (serotonin) receptor 2C	Xq23
IMPA2	Inositol(myo)-1(or 4)-monophosphatase 2	18p11.21
MLC1	Megalencephalic leukoencephalopathy with subcortical cysts 1	22q13.33
NRG2	Neuregulin 2	5q31.2
NRG3	Neuregulin 3	10q23.1
PLXNA2	Plexin A2	1q32.2
SYN2	Synapsin II	3p25.2
SYN3	Synapsin III	22q12.3
TNXB	Tenascin XB; tenascin XA pseudogene	6p21.33
TSNAX	Translin-associated factor X	1q42.2

*This list is derived from the intersection of the 1144 TargetScan putative target list with the SZGR schizophrenia-associated gene list. Cytoband location information was identified using GeneCards.

### Experimentally verified targets

Twenty-six experimentally verified targets were identified using the publicly available database TarBase (Vergoulis et al., 2012) and manual literature searches (Table 2). The functional targeting of these targets by miR-137 was confirmed by luciferase expression reporter and Western blot assays in a variety of cell lines. A PubMed search of miR-137 resulted in 44 articles, 16 of which had abstracts mentioning target gene validation experiments, which were further evaluated for their relevance. Only targets reported in articles using functional validation assays were included except for KLF4, which was confirmed by qPCR in an animal model over-expressing miR-137 (Willemsen et al., 2011). A subsequent search of hsa-miR-137 yielded no more unique articles from the previous search. Of note, ZNF804A, a gene implicated in schizophrenia in several studies (O’Donovan et al., 2008; Steinberg et al., 2011; Walton et al., 2012) was experimentally verified (Kim et al., 2012) although it was not included in the TargetScan putative target list, presumably because of the poor conservation of its binding site.

**Table 2:** **Experimentally verified targets of hsa-miR-137**.

Verified target	Assay	Cell lines	Reference	Source
CTBP1	Ago2 binding assay, luciferase assay	HEK293, A375	Deng et al. (2011)	TarBase
CDC42	Western blot, luciferase assay	SW116, Lovo, Hela, AGS, MKN45	Liu et al. (2011), Chen et al. (2011a)	TarBase
CDK6	Luciferase assay, Western blot	U251, OSCC, HEK293	Silber et al. (2008), Kozaki et al. (2008), Chen et al. (2011b)	TarBase
KDM1A (LSD1)	Luciferase assay, Western blot	HCT116, HEK293, neural stem cells	Balaguer et al. (2010), Sun et al. (2011)	TarBase, Literature search
E2F6	Western blot	OSCC	Kozaki et al. (2008)	TarBase
NCOA2	Western blot	OSCC	Kozaki et al. (2008)	TarBase
MITF	Luciferase assay, GFP reporter	HEK293, A375, WM852	Haflidadóttir et al. (2010), Chen et al. (2011b), Bemis et al. (2008)	Literature search
KDM5B (Jarid1b)	Western blot, luciferase assay	mouse ESC, HEK293	Tarantino et al. (2010)	Literature search
SPTLC1	Luciferase assay	rat primary astrocytes	Geekiyanage and Chan (2011)	Literature search
PTBP1	Luciferase assay	Neuro2a cells	Smith et al. (2011)	Literature search
CSMD1^#^	Luciferase assay	HEK-293T	Kwon et al. (2011)	Literature search
C10orf26^#^	Luciferase assay	HEK-293T	Kwon et al. (2011)	Literature search
CACNA1C^#^	Luciferase assay	HEK-293T	Kwon et al. (2011)	Literature search
TCF4^#^	Luciferase assay	HEK-293T	Kwon et al. (2011)	Literature search
CDK2	Western blot	M23 and SP6.5	Chen et al. (2011b)	Literature search
RB1 (p-Rb)	Western blot	M23 and SP6.5	Chen et al. (2011b)	Literature search
MAPK1 (p-ERK1/2)	Western blot	M23 and SP6.5	Chen et al. (2011b)	Literature search
MAPK3 (p-ERK1/2)	Western blot	M23 and SP6.5	Chen et al. (2011b)	Literature search
MET (c-Met)	Western blot	M23 and SP6.5	Chen et al. (2011b)	Literature search
ESRRA	Luciferase assay	HepG2	Zhao et al. (2012)	Literature search
PTGS2 (Cox-2)*	Western blot, luciferase assay	U87 and LN229	Chen et al. (2012)	Literature search
MIB1	Luciferase assay	DIV6 primary neurons	Smrt et al. (2010)	Literature search
MSI1	Western blot, luciferase assay	U251, Daoy, HeLa	Vo et al. (2011)	Literature search
EZH2	Luciferase assay	HEK-293T	Szulwach et al. (2010)	Literature search
KLF4	Quantitative reverse transcription PCR in over-expressing miR-137 animal model	LCL	Willemsen et al. (2011)	Literature search
ZNF804A^#^	Luciferase assay	HEK-293T, Be2C	Kim et al. (2012)	Literature search

*Indicates genes in SZGR associated list.

*^#^Indicates genes associated with schizophrenia but not in SZGR list*.

### Chromosomal location of target genes

**Figure 2:**
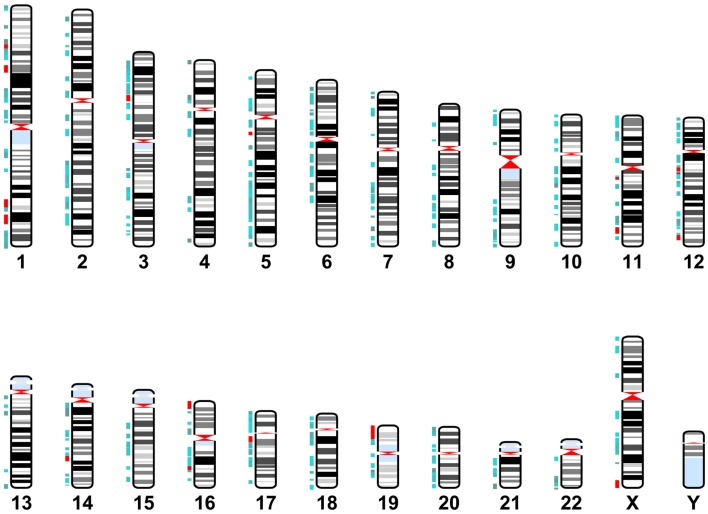
**miR-137 target gene locations**. Karyotype representing cytoband locations of the miR-137 TargetScan putative target genes. Blue depicts cytobands with two to five target genes. Red indicates regions containing six or more target genes.

Cytoband data was gathered from the UCSC database table browser, assembly dated February 2009, http://genome.ucsc.edu/ (Karolchik, 2004). miR-137 target gene location data was identified using the GeneCards database. This data was graphed using the Matlab Bioinformatics Toolbox (Figure 2). Target genes are located throughout the genome with a few localized “hot spots” (shown by red vertical lines in the figure). There are several “hotspots” located in Chromosomes 1, 11, 12, 16, and one each in chromosome 3, 14, 17, 19, and X. Comparison of the cytoband locations with miR-137 targets and those known to be affected by copy number variations (CNVs) in schizophrenia and autism spectrum disorders (ASD) (Liu et al., 2012; Sullivan et al., 2012) revealed several regions of overlap (see cytobands shown in red in Table S1 in Supplementary Material). While the proportion of overlapping cytobands did not reach global statistical significance with a hypergeometric probability test, it is important to note that one of these regions mapped to NRXN1, a 2p16.3 gene with associated deletions both in schizophrenia and ASD and whose transcript is a putative miR-137 target.

## Functional Relevance of Target Genes

### Expression of target genes

**Figure 3:**
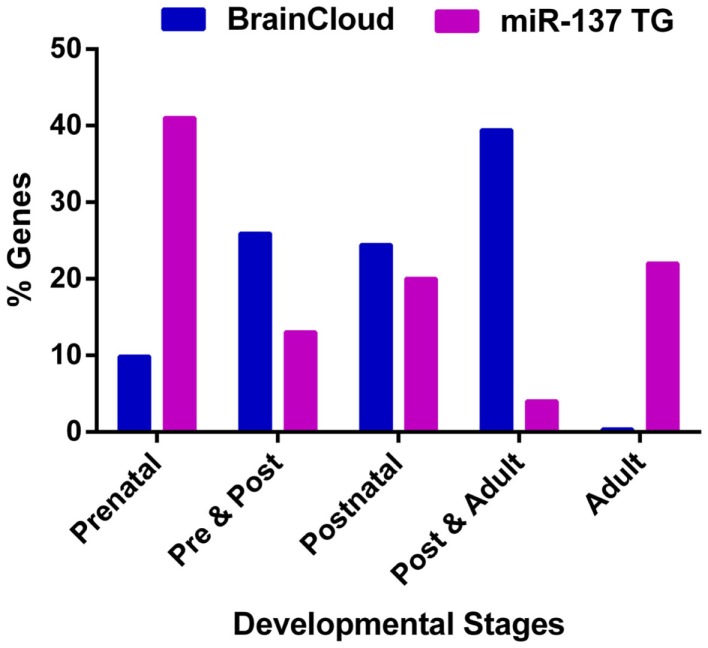
**Peak expression life stage for genes of interest**. Temporal expression data for all available miR-137 putative targets and verified targets is based on the BrainCloud Database (Kang et al., 2011). Comparison of the temporal expression profiles of miR-137 targets and whole brain transcriptome (BrainCloud) using Chi square analysis revealed that the temporal expression profile frequency distributions were significantly different (*p* < 0.01). The prenatal stage is defined as week 14 through birth, post-natal stage is defined as birth through age 20 years, and adult stage is defined as age 20 years and older.

Given that the onset of schizophrenia generally occurs in adolescence and early adulthood, we reviewed the temporal expression patterns of miR-137 target genes. Using the freely available BrainCloud expression database developed by the Lieber Institute for Brain Development (LIBD) and the National Institute of Mental Health (NIMH) (Colantuoni et al., 2011) that uses the expression profiles in the dorsolateral prefrontal cortex of human post-mortem tissue across the lifespan, we examined the expression patterns of all available experimentally verified target genes (Table 2) and putative targets common to the SZGR association list (Table 1). Except for ATXN1, CDC42, GRIN2A, KDM1A (LSD1), and SYN3 we found expression patterns for all these genes. Of the 46 examined genes, about 41% have peak expressions during prenatal life, 13% during prenatal and post-natal combined, 20% post-natal, 4% both post-natal and adult, and 22% during adulthood (Figure 3). Comparison of the patterns of expression of miR-137 targets vs. whole brain transcriptome (Kang et al., 2011) revealed that the targets have an atypical temporal distribution with peak expressions occurring more often during the prenatal period, or during adult life. The findings that 74% of target genes have peak expressions prior to adulthood, particularly prenatally, suggest that these target genes may be particularly relevant to the development of the schizophrenia. The unexpectedly large proportion of genes with peak expression in adulthood raises the possibility that miR-137 targets are involved in the ongoing decline in cognitive function and gray matter density observed in schizophrenia over the lifespan.

### Pathway analysis

Pathway analysis was performed using the putative target gene list derived from TargetScan, containing 1144 target genes and the 8 experimentally verified target genes (Table 2) not contained within the putative list. This analysis was performed in three levels using the Ingenuity Pathway Analysis (IPA) software (Ingenuity^®^ Systems, CA, USA, www.ingenuity.com) generating a canonical pathway analysis and related network analyses for each level. The networks are given a score based on the probability of inclusion of the number of molecules in the generated networks over the probability of a network being generated by chance with random molecules. This score is generated as a negative log *p* value based on a right tailed Fisher’s exact test (Calvano et al., 2005). Canonical pathway analysis is performed by comparing the dataset of interest against known canonical (signaling and metabolic) pathways within the database. A negative log *p* value is also assigned to the pathways based on a Fisher’s exact test of the probability of the number of molecules from the user-created dataset included in the given pathways vs. being included based on chance alone.

**Figure 4:**
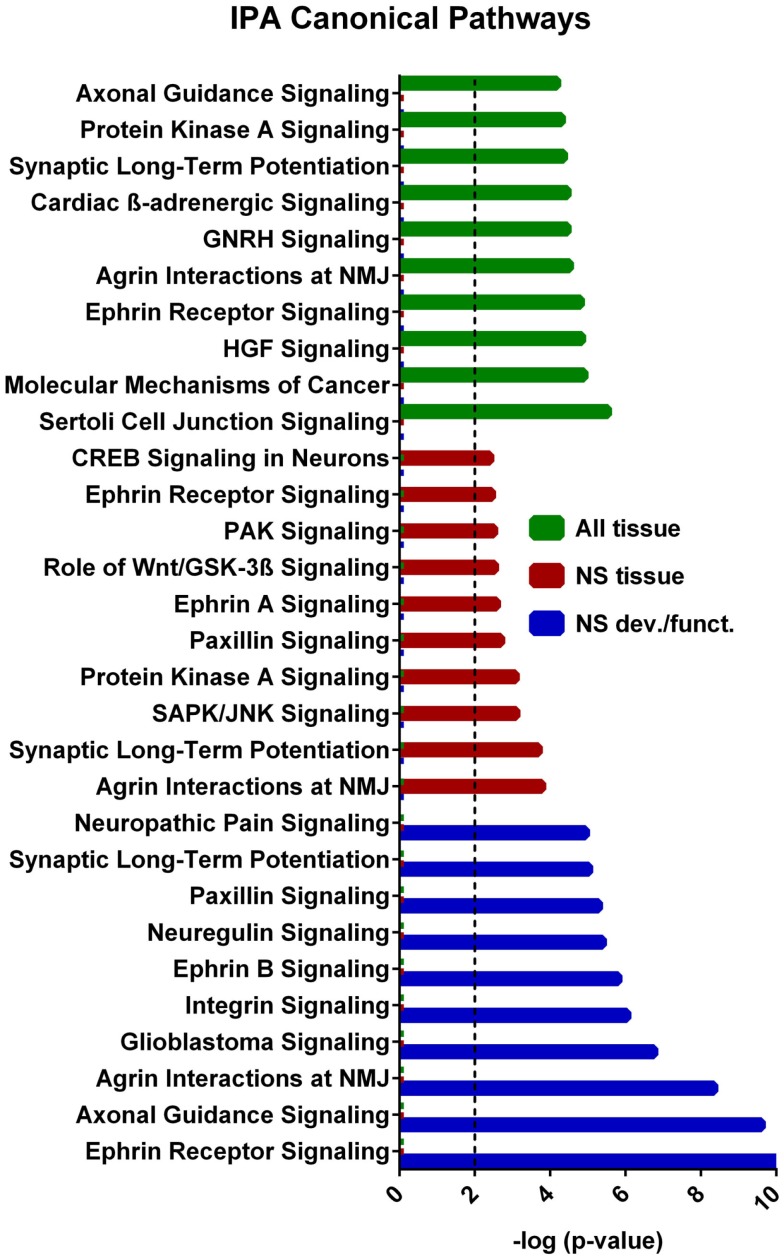
**IPA canonical pathway analyses from each tier of core analysis**. The *X* axis represents negative log p values based on the probability that molecules in the uploaded dataset were included the predefined IPA canonical pathways by true association as opposed to inclusion of molecules based on chance alone. Pathways not involved in nervous system were removed from the nervous system tissue analysis and nervous system development and function graphical displays. Only the top 10 pathways with the largest negative log *p* values are shown. The dashed line indicates the threshold of significance for a *p* value of 0.01. GNRH, gonadotropin-releasing hormone; HGF, hepatocyte growth-factor; NMJ, neuromuscular junction.

A core analysis was first performed with this data set analyzing molecules in all tissue types in mammals. Of the 1144 putative target genes, 1142 were mapped in the IPA software and usable for the analysis. In addition the eight experimentally verified transcripts not included in this putative target list were all mapped in IPA and included in the analysis, so that a total of 1150 target genes were used in the analysis. The top scoring canonical pathways corresponding to targets expressed (a) in all tissues, (b) only in the nervous system (NS), or (c) those associated with NS development and function are shown in Figure 4. Interestingly, while *Sertoli cell-signaling* (Figure S1 in Supplementary Material) was the top scoring pathway for targets expressed in all tissues, this set also included several NS-specific pathways. Among these, the top scoring pathways were *agrin interaction at the neuromuscular junction* (Figure S2 in Supplementary Material), and *synaptic long-term potentiation (LTP)* (Figure S3 in Supplementary Material). The top physiological system associated with miR-37 targets, containing 202 genes, was *NS development and function*. Using this set, we found that the top pathways were *ephrin receptor signaling* (Figure S4 in Supplementary Material), and *axonal guidance* (Figure S5 in Supplementary Material), processes known to be involved in neuronal development and cognition.

**Figure 5:**
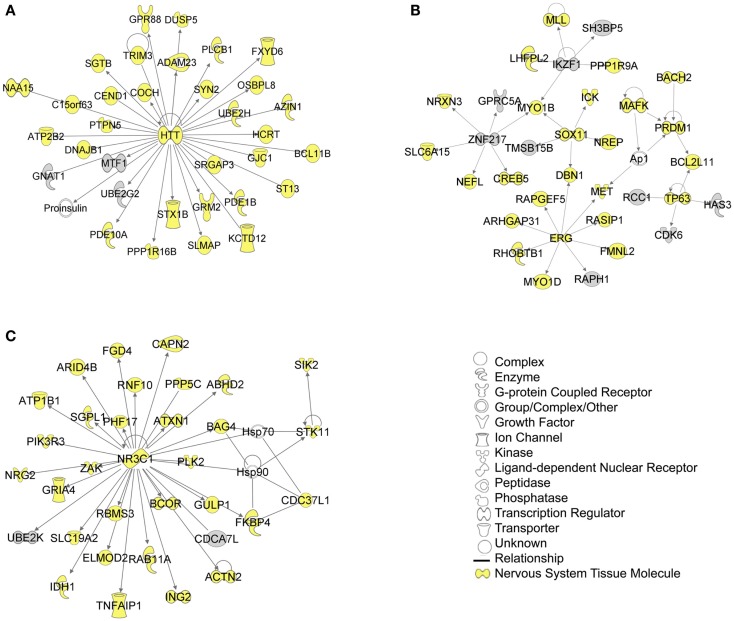
**Top 3 scoring IPA network analysis generated networks for all mapped 1150 TargetScan putative targets and verified targets**. The top 3 scoring networks identified were: **(A)** “hereditary disorder, neurological disease, skeletal and muscular disorders,” with a score of 39, **(B)** “cellular development, connective tissue development and function, cancer,” with a score of 39, and **(C)** “protein synthesis, endocrine system development and function, and lipid metabolism,” with a score of 36. Yellow indicate molecules expressed in the nervous system. White molecules are those not included in the uploaded dataset but added by IPA. Gray molecules indicate those that were included in the uploaded dataset. Relationships depicted by lines with arrows represent “act on,” while lines without arrows represent binding. See figure keys for identification of the types of molecules included.

**Figure 6:**
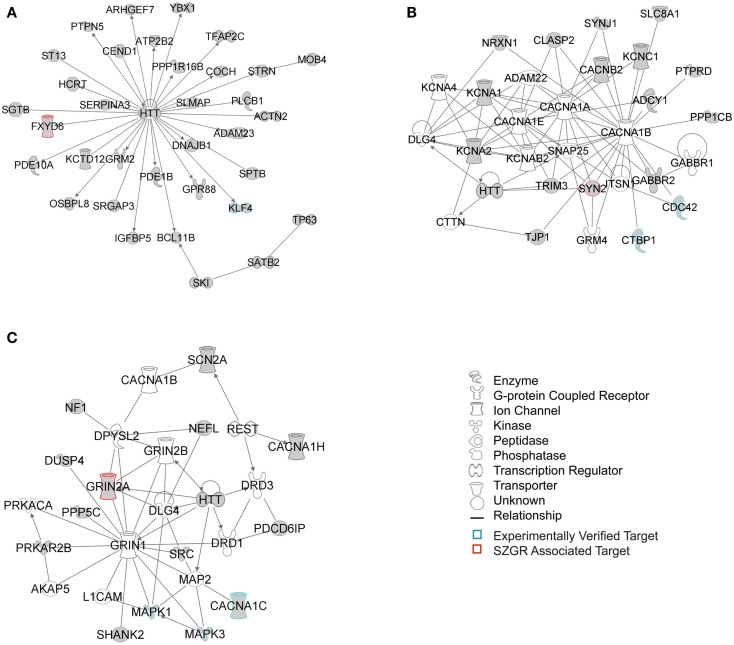
**Top 3 scoring IPA network analysis generated networks for the 929 nervous tissue specific putative and verified targets**. The top 3 scoring networks identified by this analysis were: **(A)** “hereditary disorder, neurological disease, skeletal and muscular disorders,” with a score of 35, **(B)** “neurological disease, cell-to-cell signaling and interaction, nervous system development and function,” with a score of 10, and **(C)** “behavior, cell-to-cell signaling and interaction, nervous system development and function,” with a score of 8. White molecules are those not included in the uploaded dataset but added by IPA. Gray molecules indicate those that were included in the uploaded dataset. Relationships depicted by lines with arrows represent “act on,” while lines without arrows represent binding. See figure legend keys for identification of the types of molecules included. Blue outlines depict experimentally validated targets and those in red indicate SZGR associated targets.

**Figure 7:**
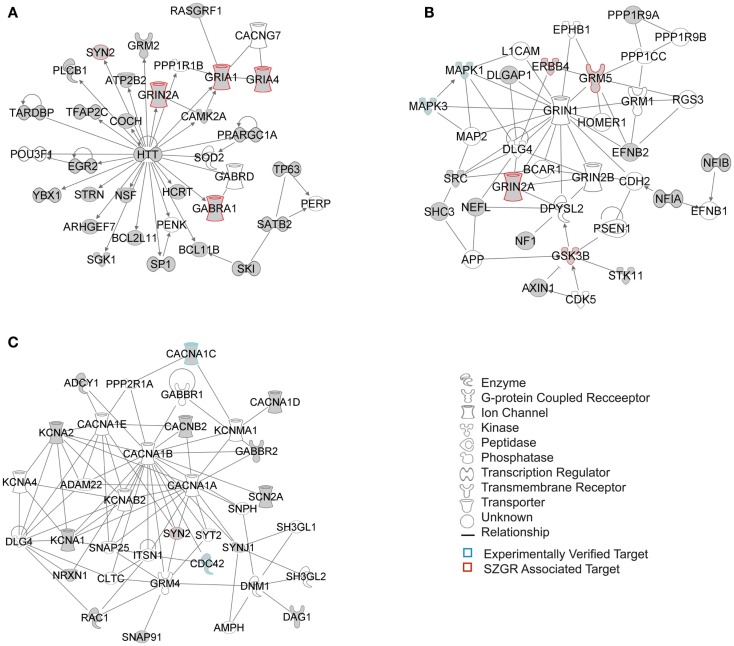
**Top 3 scoring IPA network analysis generated networks for 202 nervous system development and function subset of putative and verified targets**. The three top scoring networks identified were: **(A)** “behavior, hereditary disorder, neurological disease,” with a score of 29, **(B)** “cell morphology, cellular assembly and organization, cellular development,” with a score of 12, and **(C)** “cell-to-cell signaling and interaction, nervous system development and function, neurological disease,” with a score of 10. White molecules are those not included in the uploaded dataset but added by IPA. Gray molecules indicate those that were included in the uploaded dataset. Relationships depicted by lines with arrows represent “act on,” while lines without arrows represent binding. See figure keys for identification of the types of molecules included. Blue outlines depict experimentally validated targets and those in red indicate SZGR associated targets.

The biological significance of the molecules included in these canonical pathways is best illustrated by their associated interactive networks (Figures 5–7). As shown in Figure 5, the top networks of targets expressed in all tissues contained a large number of NS associated molecules, depicted in yellow, confirming past studies that miR-137 is involved in NS development (Silber et al., 2008; Siegel et al., 2009; Smrt et al., 2010; Szulwach et al., 2010; Sun et al., 2011). A subsequent core analysis was then performed with the 929 NS tissue associated subset of molecules of the original 1150 putative and verified target genes used in the previous analysis. This resulted in networks (Figure 6) including a substantial number of target genes found in the SZGR database and the experimentally verified list, as well as many other schizophrenia-associated genes. Finally, the top association networks of targets involved in NS development and function also contained many of the schizophrenia-associated and verified target genes (Figure 7).

## Conclusions and Perspectives

There is evidence that schizophrenia is a highly complex polygenic disorder with multiple genes contributing only a small risk. Given that individual miRNAs can affect the expression of up to thousands of genes post-transcriptionally, and that differential expression of miRNAs between patients with schizophrenia and controls has been shown in many different studies; it seems likely that miRNAs may play a role in the etiology of the disease. miR-137 in particular was shown to have a SNP (rs1625579) with the highest association with schizophrenia in the largest schizophrenia GWAS study performed to date (Ripke et al., 2011). Although the mechanisms by which this SNP may cause a dysregulation in miR-137 processing is not completely understood, recent studies suggest that carriers of the risk allele have abnormal levels of mature miR-137 in the cerebral cortex (Guella et al., 2012). Considering the biological pathways associated with miR-137 targets, there are several possible mechanisms, discussed below, by which alterations in miR-137 expression may contribute to the development and pathophysiology of schizophrenia.

Imaging genetics studies have shown an association of the miR-137 risk allele with reduced fMRI responses in both patients and at risk subjects while performing cognitive tasks (Potkin et al., 2009a,b, 2010; Whalley et al., 2012). In addition, this SNP was also shown to have a potential association with the P300 endophenotype (Decoster et al., 2012) and a significant association with cognitive deficits in patients with schizophrenia using a variety of tasks (Green et al., 2012; Cummings et al., 2013). Furthermore, a recent study (Lett et al., 2013) found that the risk allele was associated with earlier age-at-onset of psychosis, decreased hippocampal volume, and reduced white matter integrity throughout the entire brain. Given that miR-137 plays a role in neurogenesis, neurodevelopment, dendritic arborization, and is located in the synapse (Silber et al., 2008; Siegel et al., 2009; Smrt et al., 2010; Szulwach et al., 2010; Sun et al., 2011) it is enticing to propose that abnormal expression of this miRNA may lead to abnormal synapse formation which could in turn play a role in the cognitive deficits, psychotic symptoms, and brain structural abnormalities found in these patients.

By utilizing the freely accessible TargetScan, GeneCards, and UCSC databases, we found that the miR-137 potentially targets over a thousand genes, with a variety of functions and potential impact. As depicted in Figure 2 these putative target genes can be found throughout nearly every chromosome, with some particular regions of high concentration. This list of putative target genes also includes some known to be associated with schizophrenia (e.g., ERBB4, GABRA1, GRIN2A, GRM5, GSK3B, NRG2, and HTR2C) as well as many experimentally verified as true targets. Subsequent analysis of these putative and verified genes using IPA demonstrates that this list of genes includes many NS-specific genes. Expression Data from BrainCloud also confirms that many of the genes such as FXYD6, BRD1, GSK3B, CDK6, CDC42, and CACNA1C have higher expression levels in onset risk time periods, suggesting their relevance in contributing risk for development of schizophrenia. These target genes also form networks involved in neuronal function and development; with the top canonical pathways associated being *agrin interaction at the neuromuscular junction, synaptic LTP*, *ephrin receptor signaling*, and *axonal guidance signaling* (Figures S2–S5 in Supplementary Material). Sertoli cell-signaling (Figure S1 in Supplementary Material) was the top scoring pathway identified by the first tier of analysis of all 1150 putative and verified targets of miR-137, which may be related to the increased risk of schizophrenia with increasing paternal age (Frans et al., 2011). The identification of agrin interaction at neuromuscular junctions as a top canonical pathway may also be of interest given the association with neurological soft signs and schizophrenia (Sewell et al., 2010). Also, given that several of the same target transcripts play a role in cell–cell interactions in the periphery and the CNS, it is likely that this miRNA has similar roles in other tissues. However, given the enrichment of this mRNA in developing neurons, we focus our discussion in this cell type.

Many of the top scoring pathways identified: LTP signaling, ephrin receptor signaling, and axonal guidance signaling, are closely linked to learning and memory and have shown to be associated with schizophrenia. A recent study suggests evidence of impaired LTP in schizophrenia patients with a simultaneous deficit in motor learning (Frantseva et al., 2008). In addition, many schizophrenia animal model studies have also demonstrated an impairment of LTP (Pollard et al., 2012). Of particular interest, a study examining ERBB4 (one of the miR-137 putative target genes), and synaptic potentiation, demonstrated that mice with a full ErbB4 knock-out and mice with a conditional ErbB4 knock-out in only parvalbumin expressing cells exhibit increased hippocampal LTP and lack theta-pulse evoked LTP reversal (Shamir et al., 2012). These mice have increased locomotor activity in the open field test; such hyperactivity in response to novelty is used often as a model of positive symptomatology in schizophrenia. The mice also exhibit deficits in prepulse inhibition of the acoustic startle response, a test believed to model sensorimotor gating, which has been shown to be reduced in patients with schizophrenia. These findings provide further evidence that miR-137 may indeed impact LTP in schizophrenia leading to the cognitive dysfunction, a clinical feature still poorly understood and inadequately treated (Blanchard et al., 2011).

Axonal guidance has also been implicated in schizophrenia. Ephrin signaling plays a role in axonal guidance by controlling axon motility (Xu and Henkemeyer, 2012). A recent imaging study found an association between polymorphisms in RELN and PCDH12, genes involved in neuronal guidance and synaptic formation, and alterations in the patients’ brain structure as seen by MRI (Gregório et al., 2009). Another imaging study found an association with SNPs in RELN, PLXNA2, and other genes involved in axonal guidance and neuronal development and prediction of DLPFC inefficiency during a working memory task (Walton et al., 2012). There is also evidence linking ephrin receptor signaling and LTP. Particularly two studies found that post-synaptic EphB receptors and presynaptic B-ephrins are necessary for the induction of LTP of the mossy fibers in the hippocampus, a *N*-methyl-d-aspartate (NMDA) receptor independent form of LTP (Contractor et al., 2002; Armstrong et al., 2006). Of note, many genes involved in glutamate signaling such as GRIA1, GRIA4, GRIN2A, GRM2, and GRM5 were identified in the generated networks and were included in the LTP signaling cascade of the IPA canonical pathway (Figure S3 in Supplementary Material). The potential role of miR-137 in the expression of these glutamate receptors could explain not only the altered glutamate signaling observed in schizophrenia (Coyle, 2006; Sendt et al., 2012), but also the LTP disruption in the illness, which may in turn contribute to the associated cognitive deficits.

Interestingly, we also found that the Huntington’s associated protein Huntingtin (HTT) was identified as a hub in all three tiers of IPA network analyses. While the normal function of this protein is still largely unknown, it is possible that HTT has been included in the IPA database as having many biological relationships largely because it is well studied. However, recent evidence suggests that HTT is involved highly in neuronal development, playing a role in early neuronal survival, regulation of axonal transport, regulation of BDNF production particularly in the cortex, and controlling synaptic activity (Zuccato et al., 2010). Given the role of miR-137 in neuronal development these two molecules may work in concert to regulate this process. Further research will be necessary to determine if HTT plays a role in schizophrenia.

By IPA analysis of the putative and experimentally validated targets of miR-137, we identified that a large majority are expressed in the NS, forming networks involving genes associated with schizophrenia. The top canonical pathways identified by these analyses are widely known to be associated with learning and memory and synaptic formation, suggesting that the genetic impact of this miRNA may play a role in the processes of cognition and neuronal development. While our own analyses and the results of the literature support a role of miR-137 in the etiology of schizophrenia, further analysis is necessary to understand the full impact of this miRNA. In particular, it will be important to evaluate the involvement of LTP, ephrin receptor signaling, axonal guidance, and glutamate signaling. Further elucidation of the role of miR-137 in schizophrenia is merited as the negative symptoms and cognitive deficits associated are still inadequately treated and can have such a grave impact on patients. This miRNA may provide a new avenue for exploring the underlying mechanisms involved in the etiology of the disease as well as discovering new biomarkers and therapeutic targets.

## Conflict of Interest Statement

The authors declare that the research was conducted in the absence of any commercial or financial relationships that could be construed as a potential conflict of interest.

## Supplementary Material

The Supplementary Material for this article can be found online at http://www.frontiersin.org/Behavioral_and_Psychiatric_Genetics/10.3389/fgene.2013.00058/abstract

Supplementary Table S1 **miR-137 putative target list, chromosomal locations and disease associations**.

Supplementary Figure S1 **miR-137 targets in Sertoli cell-signaling canonical pathway**. Figure shows the Sertoli cell-signaling pathway from IPA with the symbols for all miR-137 putative and verified targets shown in orange.

Supplementary Figure S2 **miR-137 targets in agrin interaction at the neuromuscular junction canonical pathway**. Figure shows the agrin interaction at the neuromuscular junction pathway from IPA with the symbols for all miR-137 putative and verified targets shown in orange.

Supplementary Figure S3 **miR-137 targets in synaptic long-term potentiation (LTP) canonical pathway**. Figure shows the synaptic LTP pathway from IPA with the symbols for miR-137 putative and verified targets shown in orange.

Supplementary Figure S4 **miR-137 targets in ephrin receptor signaling canonical pathway**. Figure shows the ephrin receptor signaling pathway from IPA with the symbols for all miR-137 putative and verified targets shown in orange.

Supplementary Figure S5 **miR-137 targets in axonal guidance canonical pathway**. Figure shows the axonal guidance pathway from IPA with the symbols for all miR-137 putative and verified targets shown in orange.
