# Supplementary material for: Potential Impact of miR-137 and Its Targets in Schizophrenia
Source: Front Genet. 2013 Apr 26;4:58. doi: 10.3389/fgene.2013.00058 (PMC3636510; doi:10.3389/fgene.2013.00058)
Supplement: Supplementary Figure S1 — miR-137 targets in Sertoli cell-signaling canonical pathway. Figure shows the Sertoli cell-signaling pathway from IPA with the symbols for all miR-137 putative and verified targets shown in orange. [file 37060_Turner_Presentation1.PDF]

Supplementary materials for

Potential impact of miR-137 and its targets in schizophrenia

Carrie Wright, Jessica A. Turner, Vince D. Calhoun
and Nora Perrone-Bizzozero

This PDF includes:

Figs S1-S5

Other supplementary materials for this manuscript include:

Table S1

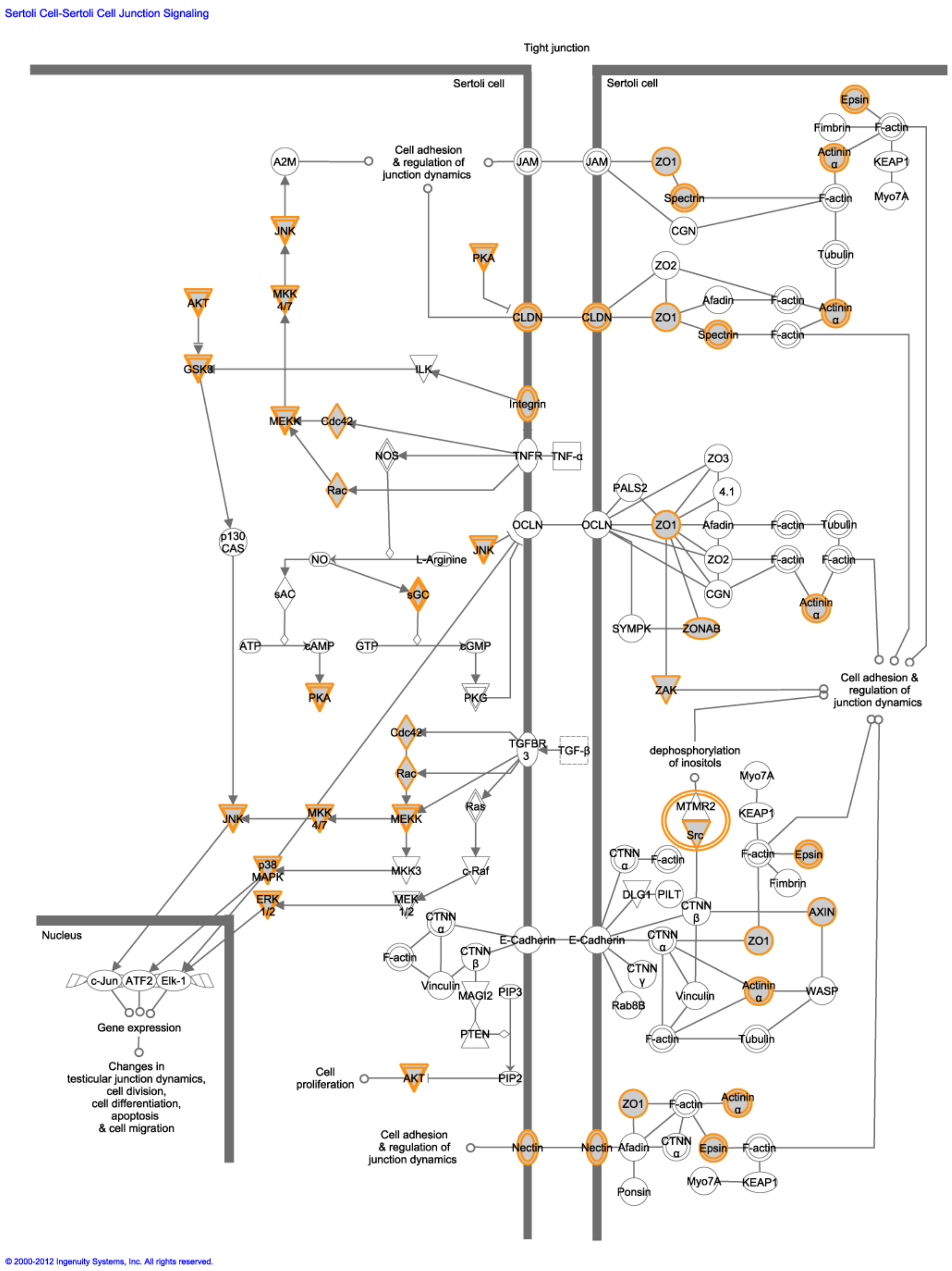

Figure S1. miR-137 targets in Sertoli cell-signaling canonical pathway. Figure shows the Sertoli cell-signaling pathway from IPA with the symbols for all miR-137 putative and verified targets shown in orange

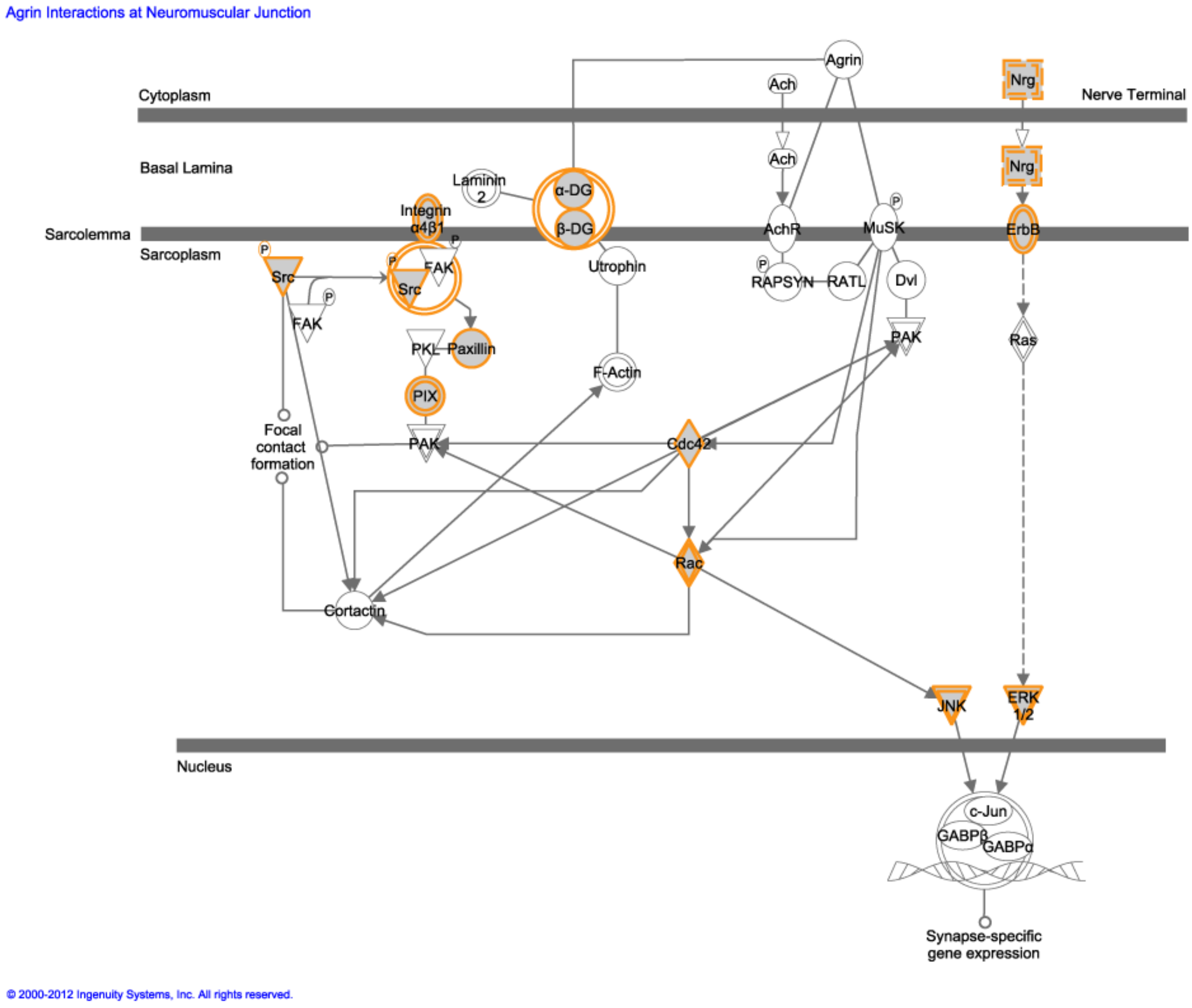

© 2000-2012 Ingenuity Systems, Inc. All rights reserved.

Figure S2. miR-137 targets in agrin interaction at the neuromuscular junction canonical pathway. Figure shows the agrin interaction at the neuromuscular junction pathway from IPA with the symbols for all miR-137 putative and verified targets shown in orange

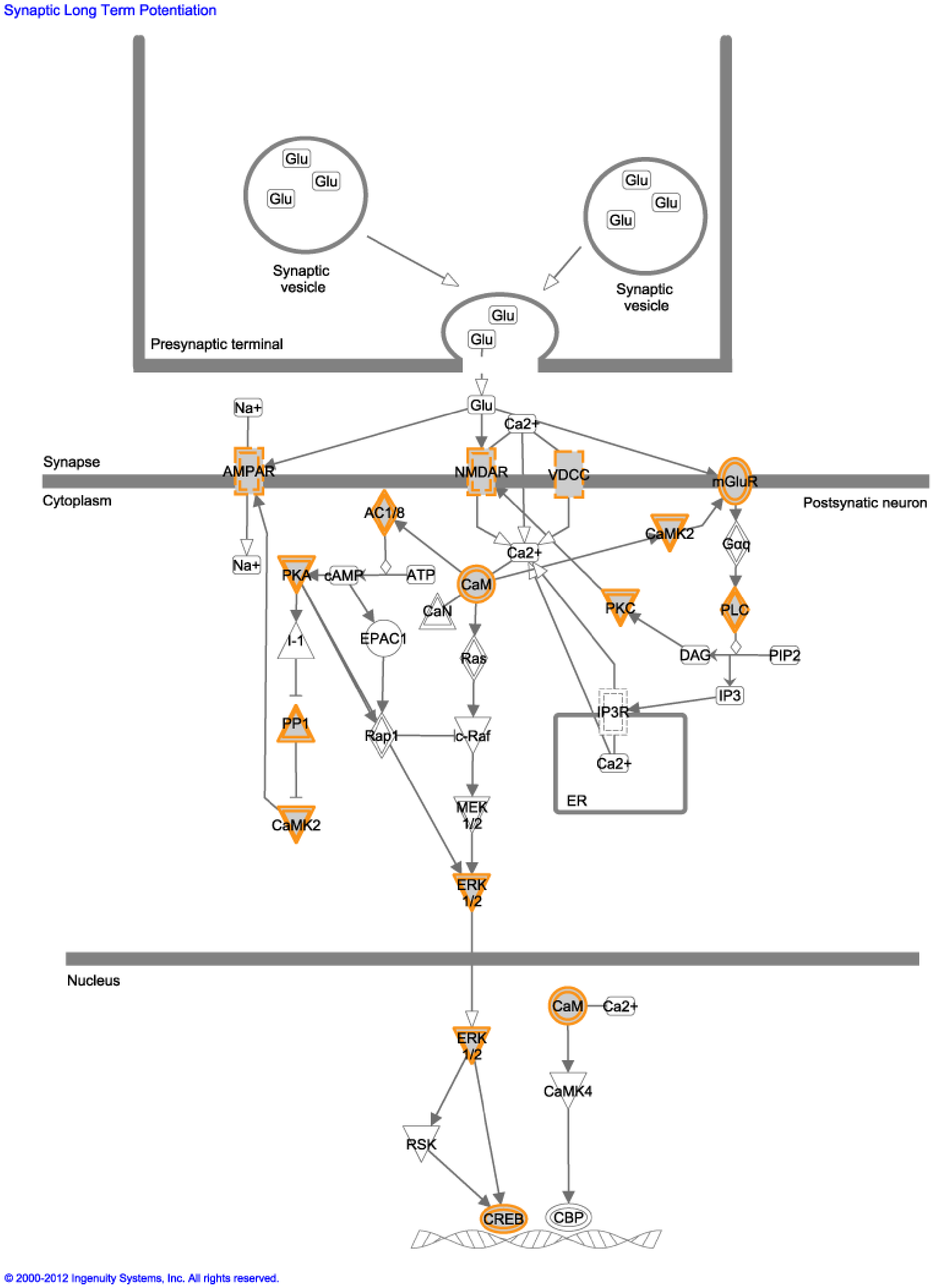

Figure S3. miR-137 targets in synaptic long-term potentiation (LTP) canonical pathway. Figure shows the synaptic LTP pathway from IPA with the symbols for miR-137 putative and verified targets shown in orange.

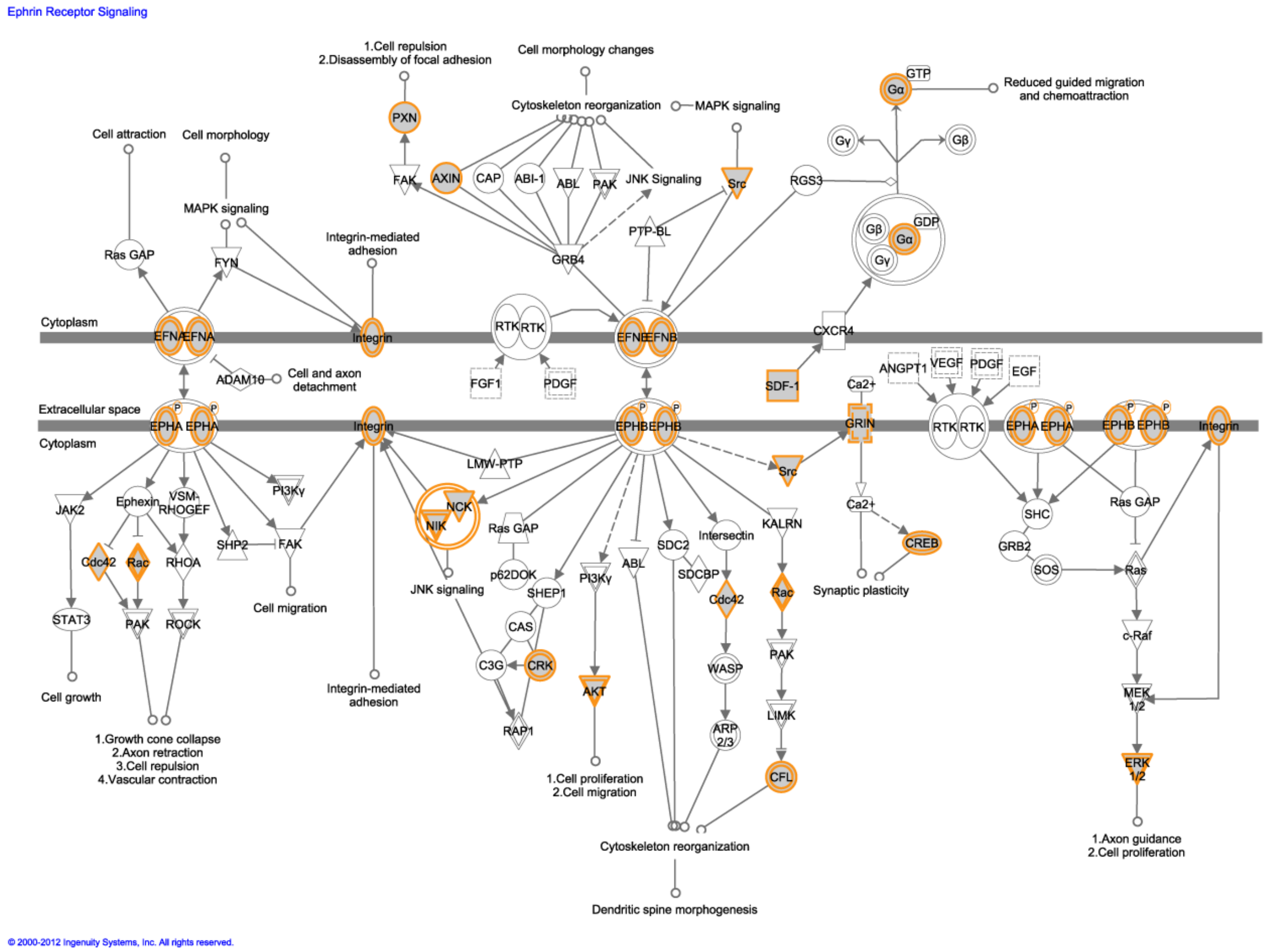

Figure S4. miR-137 targets in ephrin receptor signaling canonical pathway. Figure shows the ephrin receptor signaling pathway from IPA with the symbols for all miR-137 putative and verified targets shown in orange.

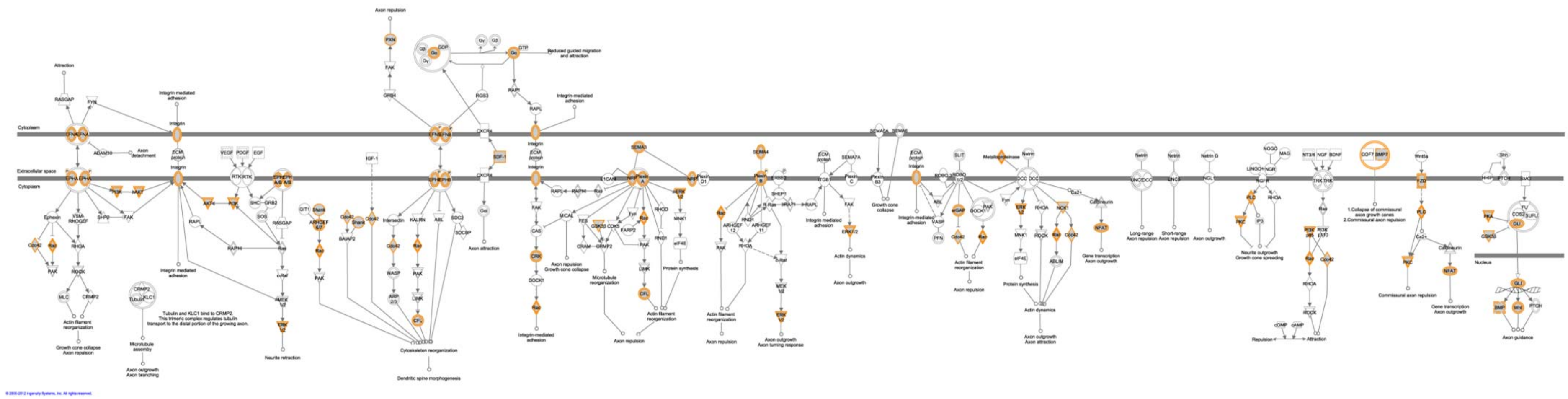

Figure S5. miR-137 targets in axonal guidance canonical pathway. Figure shows the axonal guidance pathway from IPA with the symbols for all miR-137 putative and verified targets shown in orange
